# Arginine modulates the pH, microbial composition, and matrix architecture of biofilms from caries-active patients

**DOI:** 10.1038/s41368-025-00404-5

**Published:** 2025-11-20

**Authors:** Yumi C. Del Rey, Pernille D. Rikvold, Marie B. Lund, Eero J. Raittio, Andreas Schramm, Rikke L. Meyer, Sebastian Schlafer

**Affiliations:** 1 Section for Oral Ecology, Cariology, Department of Dentistry and Oral Health, Aarhus University, Vennelyst Boulevard 9, Aarhus C, Denmark; 2 Section for Microbiology, Department of Biology, Aarhus University, Ny Munkegade 116, Aarhus C, Denmark; 3 Institute of Dentistry, University of Eastern Finland, Yliopistonranta 8, Kuopio, Finland; 4 Interdisciplinary Nanoscience Center, Aarhus University, Gustav Wieds vej 14, Aarhus C, Denmark

**Keywords:** Oral diseases, Clinical trial design

## Abstract

The caries-preventive effects of arginine have been attributed to its impact on biofilm composition and pH. Recent in vitro studies suggest that arginine also affects the production of biofilm matrix components that contribute to virulence, but this mechanism has not been investigated clinically. This randomized, placebo-controlled, triple-blind, split-mouth in situ trial assessed arginine’s impact on the microbial composition, matrix architecture, and microscale pH of biofilms from caries-active patients (*N* = 10). We also examined whether individual differences in the pH response to arginine were related to biofilm composition and matrix structure. Biofilms were grown for four days on carriers attached to intraoral splints. Three times daily, the biofilms were treated extraorally with sucrose (5 min), followed by arginine or placebo (30 min), in a split-mouth design. After growth, the microscale biofilm pH response to sucrose was monitored by pH ratiometry. Microbial biofilm composition and carbohydrate matrix architecture were analyzed by 16S rRNA gene sequencing and fluorescence lectin-binding analysis, respectively. Arginine treatment significantly mitigated sucrose-induced pH drops, reduced total carbohydrate matrix production, and altered the spatial distribution of fucose- and galactose-containing carbohydrates. Both arginine- and placebo-treated biofilms were dominated by streptococci and *Veillonella* spp. Paired analyses showed a significant reduction in mitis/oralis group streptococci and a non-significant increase in several arginine metabolizers in arginine-treated biofilms. Individual pH responses were not significantly associated with the abundance of specific bacterial taxa or carbohydrate matrix components. In conclusion, arginine reduced the virulence of biofilms from caries-active patients through multiple mechanisms, including suppressing matrix carbohydrate production.

## Introduction

The development of caries lesions is directly related to microbial acid-base metabolism in dental biofilms. The fermentation of dietary sugars by acidogenic bacteria induces pH drops, whereas the activities of alkali-producing or acid-consuming bacteria, along with salivary buffering and acid clearance, help restore physiological biofilm pH.^1,2^ Frequent sugar intake, however, favors the growth of acidogenic and acid-tolerant bacteria over the more acid-sensitive health-related organisms. This microbial imbalance and extended periods of low biofilm pH may lead to progressive dental mineral loss and, ultimately, the formation of caries cavities.^3,4^

Presently, preventive approaches that increase the resilience of dental biofilms against sugar-induced pH drops are gaining increasing interest. Arginine stimulates the proliferation and activity of organisms with the arginine deiminase system (ADS), an important alkali-producing pathway expressed by several commensal oral bacteria, including species of the genera *Streptococcus* and *Actinomyces.*^5–7^ ADS-positive bacteria utilize arginine as an energy source, and the resulting production of ammonia contributes to both intracellular and extracellular pH homeostasis.^2,8^

In vitro studies have shown that arginine metabolism raises biofilm pH and reduces demineralization by buffering microbial acid production and modulating the biofilm community structure towards less acidogenic and aciduric organisms.^9–11^ In addition, it has recently been demonstrated that arginine supplementation during in vitro biofilm growth suppresses the production of carbohydrate matrix components that may contribute to biofilm virulence.^12–14^ Data from clinical studies show promising effects of arginine treatment to prevent or arrest carious lesions, but the treatment response varies considerably between individuals and sites.^15,16^ It is conceivable that these differences are related to specific characteristics of the biofilm community structure and matrix composition, but to date, the relationship between biofilm pH response to arginine treatment and the individual biofilm composition and architecture has not been explored.

This randomized, placebo-controlled, triple-blind, split-mouth in situ study aimed to investigate the impact of arginine treatment on the microscale pH, microbial composition, and carbohydrate matrix structure of in situ-grown biofilms from highly caries-active patients. The null hypotheses were that arginine treatment does not induce changes in biofilm pH, bacterial composition and carbohydrate matrix structure, and moreover that differences in bacterial composition or matrix structure do not influence the treatment effect of arginine on biofilm pH at the individual level. The biofilm community composition was analyzed by next-generation 16S rRNA sequencing, while the presence and distribution of carbohydrate matrix components that have been previously identified as highly abundant in cariogenic biofilms were assessed by fluorescence lectin-binding analysis (FLBA). The pH response of arginine- and placebo-treated biofilms to a sucrose challenge was monitored by confocal microscopy-based pH ratiometry.

## Results

### Patient flow

**Fig. 1:**
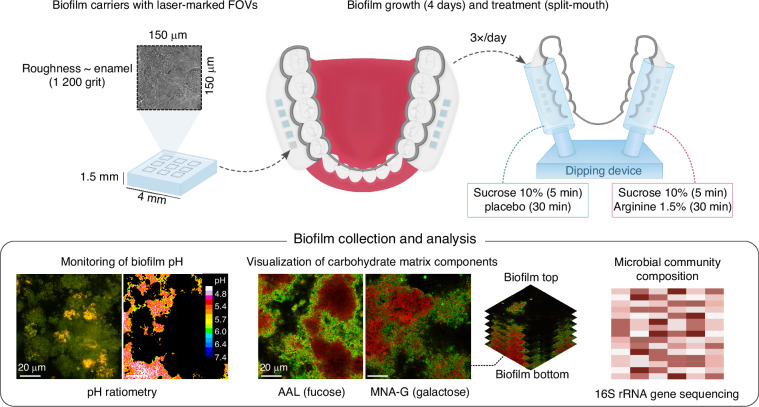
Study design. Glass slabs with a surface roughness similar to human enamel were laser-marked in nine fields of view for microscopy and inserted into individual intraoral splints to serve as carriers for in situ biofilm growth. Caries-active patients were instructed to wear the splints for 4 days of biofilm growth, during which treatment (sucrose, followed by placebo or arginine) was applied 3×/day in a split-mouth design with the aid of a dipping device. After growth, the biofilms were collected and analyzed by confocal microscopy-based pH ratiometry, to monitor the pH response of placebo- and arginine-treated biofilms to a sucrose challenge, and by fluorescence lectin-binding analysis, to visualize the distribution of fucose- and galactose-containing carbohydrate matrix components in the biofilms. Treatment-induced changes in biofilm microbial community composition were investigated by 16S rRNA gene sequencing

Twelve caries-active patients were enrolled. Participants wore individual lower-jaw splints with biofilm carriers for 4 days and dipped the splints in both sucrose and, in a split-mouth design, in either arginine or placebo solution three times per day (Fig. 1). Ten patients (M_age_ = (52.8 ± 12.8 SD) years, 3 females) completed the study protocol without reporting deviations or adverse events. Two participants were excluded for non-compliance (Supplementary Fig. 1). The average total number of active caries lesions for the included caries-active patients was 9.9 ± 5.5 SD (Supplementary Table 1).

### Arginine mitigates sucrose-induced biofilm pH drops

**Fig. 2:**
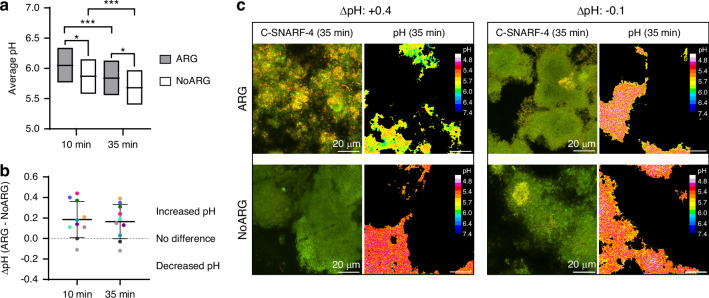
Effect of arginine treatment on biofilm pH. **a** Average extracellular pH of arginine-treated (ARG) and placebo-treated (NoARG) biofilms after 10 and 35 min of sucrose challenge. Biofilm pH dropped significantly over time for both groups (***P < 0.001), and ARG biofilms exhibited a higher average pH at both time points (*P < 0.05). Bars show means and the upper and lower limits of 95% confidence intervals. **b** Paired pH differences (ΔpH) between ARG and NoARG biofilms show that not all individuals responded equally well to arginine treatment. The colored dots represent the average ΔpH of duplicate biofilms for each patient (*N* = 10) at both time points; lines indicate the mean ΔpH (10 min: 0.19; 35 min: 0.16). **c** Representative C-SNARF-4 images and the corresponding color-coded images of extracellular pH illustrate the effect of arginine on biofilm pH of a good treatment responder (left panels, ARG: pH 5.9, NoARG: pH 5.5, ΔpH: +0.4) and a non-responder (right, ARG: pH 5.4, NoARG: pH 5.5, ΔpH: -0.1)

Biofilms treated with placebo (NoARG) were slightly thicker than biofilms treated with arginine (ARG) (31.60 ± 14.88 SD) µm vs. (24.71 ± 14.75 SD ) µm; *P* = 0.053). The biofilm pH response was monitored ratiometrically in different fields of view (FOVs) at the biofilm base, 10 min and 35 min after exposure to sucrose (4% w/v). ARG biofilms showed a higher pH than NoARG biofilms after both 10 min (6.05, 95% CI: 5.77–6.34 vs. 5.87, 95% CI: 5.58–6.15; *P* = 0.014) and 35 min (5.84, 95% CI: 5.56–6.13 vs. 5.68, 95% CI: 5.40–5.97; *P* = 0.037) of sucrose exposure (Fig. 2a). For both groups, biofilm pH dropped significantly over time (*P* < 0.001).

Biofilm pH levels and the treatment response to arginine varied considerably among individuals (Fig. 2b; Supplementary Fig. 2). For most participants, the mean pH difference (ΔpH) between paired ARG and NoARG biofilms was positive (10 min: range 0.11–0.44; 35 min: range 0.13–0.39); two subjects, however, did not respond to treatment (ΔpH≤0; Fig. 2b). Figure 2c illustrates the typical pH response of ARG and NoARG biofilms to sucrose challenge for a good treatment responder and a non-responder. pH levels also varied considerably between FOVs of the same biofilm (Supplementary Table 4). The largest differences observed between FOVs of the same biofilm were 0.70 (10 min) and 0.78 pH units (35 min) for ARG biofilms, and 0.77 (10 min) and 1.30 units (35 min) for NoARG biofilms.

### Arginine modulates the biofilm microbial community composition

**Fig. 3:**
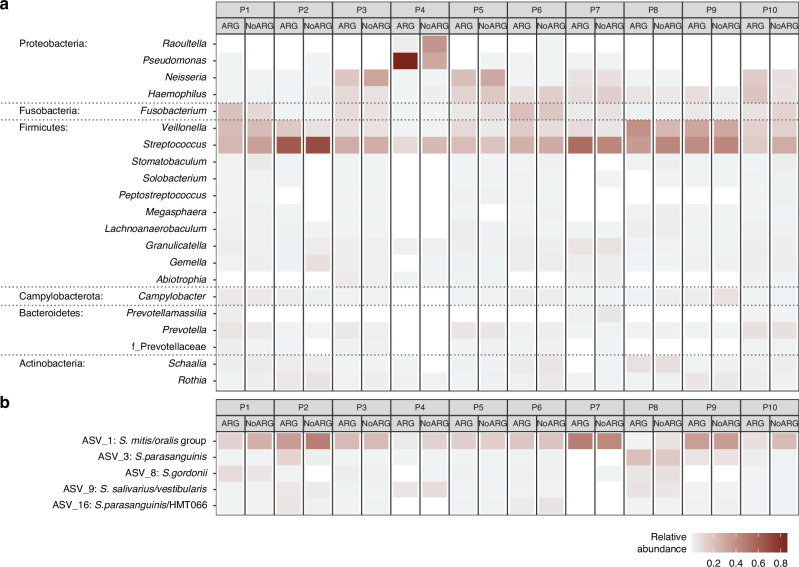
Microbial community composition of biofilms treated with arginine (ARG) or placebo (NoARG). **a** Heatmap of the most abundant bacterial genera (mean relative abundance cutoff = 1%) in ARG and NoARG biofilms from each caries-active patient (P1-P10). **b** Heatmap showing all *Streptococcus* ASVs with mean relative abundances >1% across groups. ASV1, with 100% sequence identity to the mitis/oralis group, was significantly more abundant in NoARG than in ARG biofilms

Microbial composition analysis, performed by next-generation sequencing, revealed a community structure typical for four-day-old dental biofilms. Both groups were dominated by *Streptococcus* spp. (mean relative abundances of 32.2% ± 16.8% SD for ARG and 36.1% ± 14.5% SD for NoARG) and *Veillonella* spp. (mean relative abundances of 16.9% ± 11.6% SD for ARG and 12.3% ± 9.9% SD for NoARG), with lower contributions of *Neisseria* spp., *Fusobacterium* spp., and *Haemophilus* spp. (mean relative abundances ranging from 6.2% to 7.8% across groups; Fig. 3a). Differential abundance analysis of paired ARG and NoARG biofilms identified 26 genera and 169 ASVs that differed significantly between groups. *Rothia spp*. were more abundant in NoARG than in ARG biofilms, while the opposite was observed for *Granulicatella* (*P* < 0.05); all other differentially abundant genera had mean overall abundances <1% (Supplementary Table 3).

**Fig. 4:**
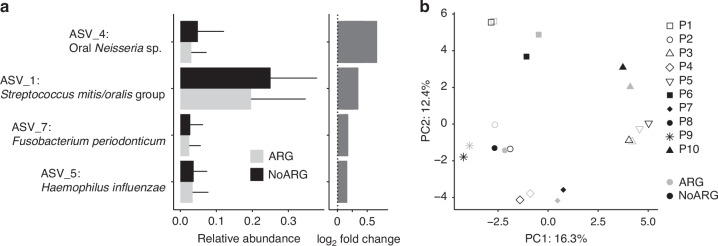
Differential abundance analysis between biofilms treated with arginine (ARG) and placebo (NoARG). **a** Mean relative abundance (± SD) and log2-fold differences in abundance between ARG and NoARG biofilms of differentially abundant ASVs (mean relative abundance cutoff = 1%). Positive log2-fold change values = higher abundance in NoARG biofilms. All paired differential abundance analyses were performed using the Wilcoxon signed-rank test, with a false discovery rate-adjusted *P*-value threshold of <0.05. ASVs were assigned to species using the Basic Local Alignment Search Tool (BLAST) of the expanded Human Oral Microbiome Database (eHOMD, http://www.homd.org). **b** Principal component analysis (PCA) showed that samples clustered according to patient, rather than treatment group

The most abundant streptococcal ASVs in both groups showed 100% sequence identity to *Streptococcus mitis/oralis* group (ASV1), *Streptococcus parasanguinis*/HMT066 (ASV16), *S. parasanguinis* (ASV3), *Streptoccocus salivarius/vestibularis* (ASV9), and *Streptococcus gordonii* (ASV8) (Fig. 3b, Supplementary Table 2). Species with high arginolytic potential^5^ (*S. parasanguinis* and *S. gordonii*) were more abundant in ARG biofilms, while the opposite trend was observed for ASV1 (mitis/oralis group) and ASV9 (*S. salivarius/vestibularis*). Those differences, however, were only significant for ASV1 (ARG: 19.6% ± 15.2% SD; NoARG: 25.0% ± 12.9% SD). Other significantly differentially abundant ASVs included ASV7 (100% sequence identity with *Fusobacterium periodonticum*), ASV5 (*Haemophilus parainfluenzae*), and ASV4 (oral *Neisseria sp.)*, which were more abundant in NoARG biofilms (Fig. 4a).

Principal component analysis showed a considerably higher compositional similarity between samples from the same patient than between samples from the same treatment group (Fig. 4b). No significant correlation between the effect of arginine on biofilm pH and bacterial biofilm composition was observed (Supplementary Table 5).

### Arginine impacts the production and distribution of biofilm carbohydrate matrix components

Fucose- and galactose-containing carbohydrate matrix components, which were shown to be highly abundant in biofilms grown under cariogenic conditions,^17,18^ were visualized by FLBA using the lectins AAL and MNA-G, and quantified by confocal microscopy and digital image analysis. The typical binding patterns observed for each lectin are illustrated in Supplementary Fig. 3. Biofilms from one participant could not be imaged due to poor sample quality (high number of epithelial cells).

**Fig. 5:**
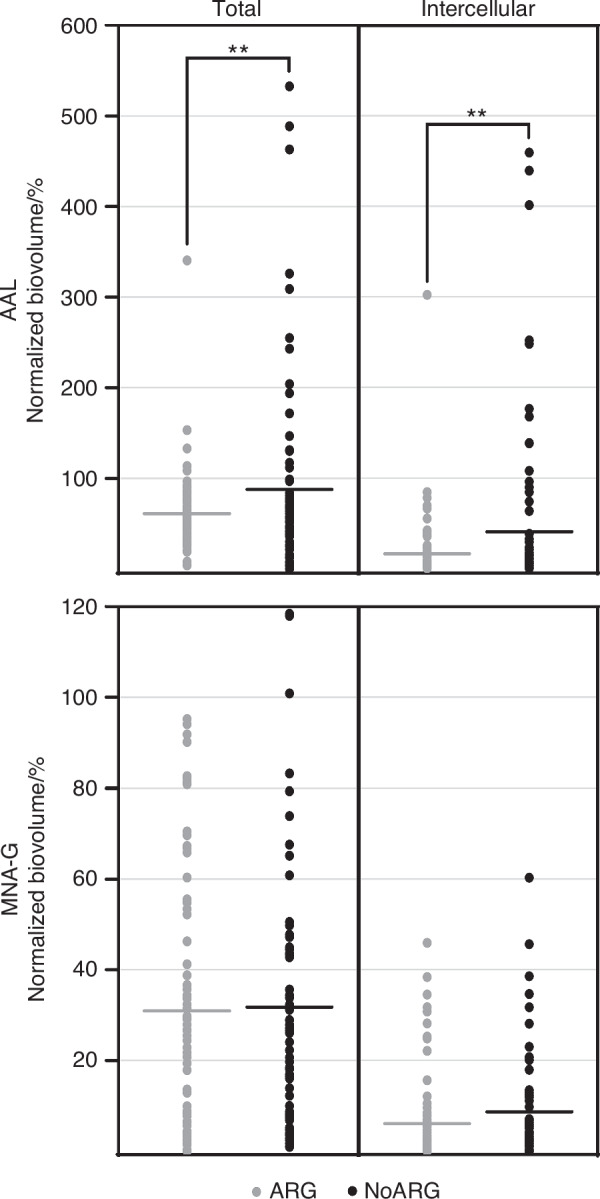
Abundance of carbohydrate matrix components in biofilms treated with arginine (ARG) or placebo (NoARG). Arginine treatment significantly reduced the total and intercellular amounts of fucose-containing matrix components (stained by AAL), but it had no effect on the abundance of galactose-containing matrix components (stained by MNA-G). Dashes show the mean normalized biovolume (% of the microbial biovolume) of AAL- and MNA-G-stained matrix components; dots represent individual fields of view in the biofilms (*N* = 9 FOVs per biofilm; *N* = 9 biofilms per lectin and group)

**Fig. 6:**
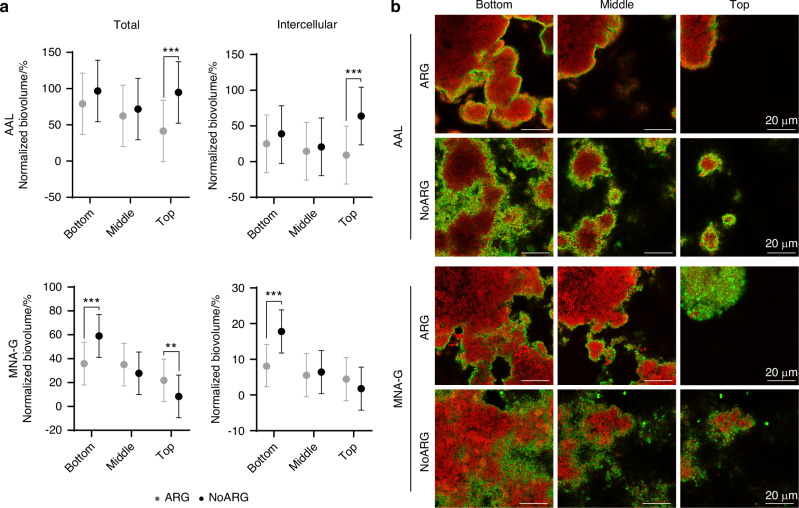
Distribution of carbohydrate matrix components in biofilms treated with arginine (ARG) or placebo (NoARG). **a** AAL targeted lower amounts of total and intercellular matrix components in all layers of ARG compared to NoARG biofilms, with the difference reaching significance for the biofilm top. For MNA-G-targeted matrix carbohydrates, arginine treatment changed the distribution across biofilm layers. ARG biofilms exhibited significantly decreased total and intercellular amounts of MNA-G-stained matrix components at the biofilm bottom and significantly increased the total amounts at the biofilm top compared to NoARG. Dots and bars show the estimated means and lower and upper limits of 95% confidence intervals. **b** Representative images of lectin-stained areas (green) and microbial cells (red) in the different biofilm layers for ARG and NoARG biofilms. **P < 0.01, ***P < 0.001

Arginine treatment reduced the amount of AAL-stained (fucose-containing) matrix components compared to placebo treatment. Significantly lower total and intercellular biovolumes were observed for the entire biofilm biovolume (*P* = 0.009 and 0.007, respectively) and in the top layer of the biofilm (*P* = 0.000 5 and 0.000 1, respectively) (Figs. 5 and 6; Supplementary Table 6).

The biovolume of MNA-G-stained (galactose-containing) matrix components was not affected by arginine treatment (Fig. 5; total: *P* = 0.78; intercellular: *P* = 0.06). However, the distribution of MNA-G-stained carbohydrates across biofilm layers changed significantly. Compared to NoARG, ARG biofilms exhibited significantly lower total and intercellular amounts of MNA-G-stained matrix components at the biofilm bottom (total and intercellular: *P* < 0.000 1) and significantly higher total amounts in the top layer (*P* = 0.001) (Fig. 6; Supplementary Table 6).

At the individual level, no relationship between the effect of arginine on biofilm pH and the respective total and intercellular AAL- and MNA-G-targeted matrix biovolumes was found (Supplementary Fig. 4). Normalized lectin-stained biovolumes varied considerably between different FOVs of the same biofilm (Supplementary Table 4). The highest observed differences in normalized lectin-stained biovolumes between FOVs within the same biofilm were 601% (NoARG) and 333% (ARG) for AAL, and 80% (NoARG) and 70% (ARG) for MNA-G.

## Discussion

The present study is the first to evaluate the combined effects of arginine treatment on biofilm composition, carbohydrate matrix architecture, and microscale pH of biofilms from caries-active patients. The first null hypothesis that arginine does not impact biofilm pH, composition, and carbohydrate matrix architecture was rejected. Arginine treatment during biofilm growth enhanced biofilm resilience against sucrose-induced pH drops, and it changed both the bacterial composition and the composition and distribution of matrix carbohydrates. Some of the changes observed in the bacterial biofilm composition are readily explained by the action of arginine, such as the reduction of mitis/oralis group streptococci, which typically exhibit low ADS activity, and the increase of *S. parasanguinis* and *S. gordonii*, both of which have been shown to possess high arginolytic potential.^5^ The overall composition of the biofilms, however, was more influenced by the factor patient than by arginine or placebo treatment, which is consistent with previous reports on the human oral microbiota.^19,20^

FLBA visualizes specific glycoconjugates and/or polysaccharides attached to cell surfaces or present in cell-free areas of the biofilms (intercellular carbohydrates; Supplementary Fig. 3).^21^ Both the total and intercellular amounts of AAL-targeted (fucose-containing) matrix carbohydrates were significantly reduced in ARG biofilms, which aligns with the results of an in vitro study that showed a strong reduction induced by arginine in 3-day-old saliva biofilms.^22^ The observed reduction may either be caused by a suppressive effect of arginine on bacterial matrix carbohydrate production, or else be a secondary effect of arginine-induced changes in the microbial community composition.^12,14^ ARG biofilms showed a non-significant trend towards reduced intercellular MNA-G-targeted carbohydrates, but no difference in the total amount of carbohydrates. Interestingly, arginine treatment significantly reduced the amount of MNA-G-targeted carbohydrates at the biofilm base, while increasing the amount at the biofilm top. It is conceivable that arginine suppresses the production of MNA-G-targeted (galactose-containing) carbohydrates by early colonizers, which predominate at the biofilm bottom, but has the opposite effect during later stages of biofilm growth.

To date, very little is known about fucose- and galactose-containing matrix carbohydrates, despite evidence of their high abundance in dental biofilms.^17,18,21^ Fucose and galactose are some of the main sugars in the carbohydrate chains of salivary mucins. Many oral organisms, in particular streptococci, possess enzymes that cleave these chains, releasing sugars that serve as nutrients for bacterial growth and, possibly, for matrix production.^23,24^ This has been demonstrated in biofilms formed by *Klebsiella pneumoniae*, which utilizes the fucose gained from the breakdown of gastrointestinal mucins for growth and biofilm matrix production.^25^ Extracellular fucosidase and galactosidase activities have been identified in oral streptococci from the mitis/oralis group,^26–28^ and these taxa exhibited a higher abundance in control-treated biofilms (ASV1, Fig. 4). We therefore hypothesize that arginine treatment reduced the abundance of acidogenic *S. mitis/oralis* in the biofilms, which in turn resulted in reduced degradation of salivary mucins and decreased production of fucose- and galactose-containing matrix carbohydrates.

The second null hypothesis of this study, namely that differences in microbial or matrix composition do not influence arginine’s treatment effect on biofilm pH at the individual level, could not be rejected. The study participants responded differently to arginine treatment (differences between treatment and control side ranging from -0.12 to 0.39 pH units), but there was no association between a participant’s biofilm pH response and the abundance of fucose- or galactose-containing matrix components. The same was true for an individual’s pH response and the abundance of specific genera or ASVs. The sample size calculation for the present study was performed based on a previous in vitro study that assessed microscale pH in a salivary biofilm model,^22^ but the variability between in situ biofilms was higher and thereby reduced our ability to detect statistically significant differences. Moreover, it is important to note that different biofilm carriers were used for pH ratiometry, FLBA, and 16S rRNA gene sequencing for each patient. Therefore, regression analyses between pH response and matrix or bacterial composition could only be performed using patient averages. Our study revealed considerable variation in pH and matrix biovolumes between different FOVs within the same biofilm, and it has been previously shown that the local biofilm architecture has a dramatic influence on the development of pH microenvironments in dental biofilms.^1,29,30^ Future studies should therefore investigate how the effect of arginine treatment on microscale pH depends on the local bacterial and matrix composition. Methods for the coupled analysis of biofilm pH, microbial composition, and carbohydrate matrix architecture at the FOV level have been recently developed,^18,31^ which may provide further insight into the effect of caries-preventive therapies at the microscale.

This study investigated the abundance of fucose- and galactose-containing carbohydrate matrix components, but arginine treatment may also affect the production of other matrix carbohydrates, such as dextrans or mutans. Lectins with high affinity for glucose residues (e.g., Concanavalin A), however, have been shown not to bind effectively to dental biofilms^21^ and were therefore not included in this study. Future work may substantiate our FLBA-based findings using complementary methods, such as high-performance liquid chromatography^32^ or gas chromatography-mass spectrometry,^33^ to investigate the production of distinct matrix carbohydrates.

In conclusion, arginine treatment increased the resilience of biofilms against sucrose-induced pH drops and modulated the biofilm microbial composition and matrix architecture of in situ-grown biofilms from highly caries-active patients. Biofilm pH response to arginine treatment varied considerably between patients, but these differences could not be linked to specific differences in the microbial community composition or the carbohydrate matrix architecture at the patient level. Our results revealed considerable variation in pH response and carbohydrate matrix production, not only at the individual level but also between FOVs within the same biofilms. Future studies may further explore the interplay between arginine treatment response and biofilm architecture by applying combined methods for the microscale analysis of pH, biofilm composition, and matrix structure.

## Materials and methods

### Study design

Twelve caries-active patients were enrolled in this randomized, placebo-controlled, triple-blind (participants, investigators, and outcome assessors), split-mouth in situ trial (Fig. 1). The study was conducted in accordance with the Declaration of Helsinki and its amendments and approved by the Ethical Committee of Region Midtjylland (1-10-72-218-22). The study protocol was deposited on clinicaltrials.gov (NCT06135792). Written informed consent was obtained from all participants.

### Sample size and study participants

Sample size calculation was based on the results of an in vitro study investigating the effect of arginine treatment on the pH of saliva-derived biofilms.^22^ Calculations were performed using the difference in hydrogen ion concentrations [H ^+^ ] between arginine- and placebo-treated biofilms (mean difference in [H^+^] of 1.03 × 10^−7^ mol/L, pooled SD of 1.39 × 10^−7^ mol/L). Using α = 0.05 and β = 0.20, a sample size of *N* = 7 was estimated. To account for the increased variability in clinical samples and potential dropouts, twelve caries-active patients were enrolled. Inclusion criteria were the presence of three or more active carious lesions (cavitated or non-cavitated), and the presence of enough teeth to retain an intraoral lower-jaw splint. Exclusion criteria were patients with severe systemic diseases, individuals who received anti-inflammatories or antibiotics within the past 3 months prior to enrollment, nursing or pregnant women, and users of retainers, orthodontic appliances, or removable dentures.

### Intraoral splints and laser-marked biofilm carriers

Splints for in situ biofilm growth were fabricated for each participant from digital impressions obtained using an intraoral scanner (TRIOS4; 3Shape, Copenhagen, Denmark), as described by Rikvold et al.^34^. Briefly, each splint consisted of a half-round metallic bar (1.75 × 1.38 mm^2^) and two buccal flanges with 3-D-printed inserts produced by vat photopolymerization (Asiga MAX UV; Alexandria, Australia). Each insert was designed to carry five biofilm carriers (custom-made non fluorescent glass slabs, 4 × 4 × 1.5 mm^3^, surface roughness ~ human enamel: 1 200 grit; Menzel, Braunschweig, Germany) with a standardized 3 mm recession depth (Fig. 1). Prior to experimental use, the carriers were marked in nine fields of view (FOVs) using a laser microdissector (Leica LMD7000; Leica Microsystems, Wetzlar, Germany) to standardize the imaged biofilm areas. The marks were placed under 20x magnification using the following laser settings: power 60, speed 10, balance 15, aperture 30, head current 70%, and pulse frequency 500. The FOVs were 150 µm × 150 µm in size, placed 500 µm apart from each other, and at least 1 200 µm away from the corners of the biofilm carriers.

### Biofilm growth and study intervention

For in situ biofilm growth, the participants were instructed to wear the intraoral splints for 4 days, and to remove them only during meals, the intake of drinks other than water, when performing oral hygiene procedures, and during biofilm treatment. Outside the mouth, the participants kept the splints in a humid chamber to prevent dehydration of the biofilm samples. Biofilm treatment was performed 3×/day. First, the participants dipped the splint into 10% (w/v) sucrose solution (5 min) to provide additional nourishment to the growing biofilms. Immediately after the sucrose dip, one side of the splint was immersed into the treatment solution and the other side into the placebo solution (30 min). Arginine treatment solution (1.5 w/v%) was prepared by dissolving L-arginine (99%, FCC, FG, W381918; Sigma Aldrich, Missouri, USA) in distilled water, followed by filter-sterilization (0.22 µm, polyethersulfone membrane; Biofil Chemicals and Pharmaceuticals Ltd., Indore, India). Sterile distilled water was used as the placebo control. Dipping devices were fabricated to aid the immersion of the growing biofilms in sucrose, and to allow for simultaneous dipping of the buccal flanges into fresh treatment or placebo solutions.

Participants received written and oral instructions on how to perform the interventions and documented their compliance in the provided sheets. The side of the splint that was immersed into the treatment solution was pre-determined prior to patient enrollment by random permutation with a block size of 2. Treatment allocation was only revealed when data analysis was complete. After growth, the in situ biofilms were collected and analyzed by pH ratiometry, fluorescence lectin-binding analysis (FLBA), or 16S rRNA gene amplicon sequencing. The study design and interventions are illustrated in Fig. 1.

### Calibration of C-SNARF-4 for pH ratiometry

The pH response of ARG and NoARG biofilms to sucrose challenge was monitored using the ratiometric dye C-SNARF-4 (Thermo Fisher Scientific, Roskilde, Denmark), which exhibits a pH-dependent shift in fluorescence that can be exploited to measure extracellular pH in biofilms.^35^ Dye calibration was performed using MES buffer solutions (Sigma-Aldrich), titrated from pH 4.0-8.0 in steps of 0.2 pH units, as described in detail elsewhere.^18^ Briefly, images of each buffer solution (50 µmol/L) mixed with C-SNARF-4 (30 µmol/L) were acquired in three randomly chosen FOVs in a 96-well microplate (µ-plate 96-well square; Ibidi, Munich, Germany) using an inverted confocal microscope (Zeiss LSM 700; Zeiss, Jena, Germany) equipped with a 63x/1.40 oil-immersion objective (alpha Plan-Apochromat, Zeiss). The dye was excited at 555 nm and the fluorescence emission was detected simultaneously from 300-618 nm (green channel) and 618-800 nm (red channel) using the following parameters: pinhole size 1.76 AU (1.3 µm optical slice thickness), image size 512 × 512 pixels (101.61 × 101.61 µm^2^), pixel dwell time 12.61 µs, and 8-bit intensity resolution. Based on the dye calibration, the following Eq. (1) was obtained using the MyCurveFit Data Analysis Tool (My Assays Ltd., Brighton, UK) to convert fluorescence emission ratios to pH values. In Eq. (1), the variable *r* represents the ratio between the fluorescence intensities detected in the green and red channels:

1 $${pH}={\left(\left(\left(\frac{2.249}{r-0.171}\right)-1\right)\times 136\,911\,185\,393\right)}^{\frac{1}{14:531\,78}}$$

### Confocal microscopy-based pH ratiometry

In situ-grown biofilms collected from each patient (*N* = 2 per intervention) were washed with 400 µL of sterile saline solution (pH 7.0; 15 min) to neutralize biofilm pH. Subsequently, the biofilms were exposed to a sucrose challenge by individually placing each glass slab on top of a coverslip with 20 µL of saline solution (pH 7.0) containing sucrose (4% w/v) and C-SNARF-4 (30 µmol/L). Biofilm pH developments were monitored in the laser-marked FOVs after 10 and 35 min of sucrose exposure. Paired ARG and NoARG biofilms were analyzed in parallel. All FOVs were imaged at the biofilm-substrate interface (biofilm bottom) using the same settings as for dye calibration.

**Fig. 7:**
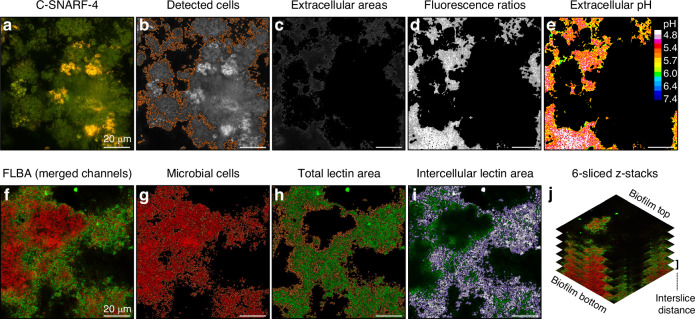
Digital image analysis procedures for pH ratiometry (top) and fluorescence lectin-binding analysis (FLBA, bottom). **a** Biofilms were imaged with the ratiometric dye C-SNARF-4 after exposure to a sucrose challenge. Fluorescence signals were detected simultaneously in two different channels (red, green). **b**, **c** Using intensity-based threshold segmentation, the microbial cells were detected in all images (outlined in orange) and subsequently removed, leaving only extracellular biofilm areas. **d**, **e** Fluorescence intensity ratios between green and red channel images were calculated for the extracellular areas and converted to pH values using a calibration equation. The corresponding color-coded image illustrates the fluorescence ratios converted to pH values. **f** FLBA images (6-sliced z-stacks) were obtained using the fluorescently-labeled lectins AAL or MNA-G (green). Microbial cells were counterstained with SYTO60 (red). **g**, **h** The areas covered by bacteria (red, outlined in orange) and by lectins (green, outlined in orange) were calculated in all images using intensity-based thresholding. **i** The intercellular lectin areas were calculated by excluding areas associated with microbial cells from the total lectin area, as shown in the superimposed image (white: intercellular lectin areas, green: lectins associated with bacterial cell surfaces). **j** Biovolumes of microbial cells and lectin-stained matrix components in the different layers of the biofilms, as well as the total biofilm biovolumes (slices 1–6), were calculated by multiplying the respective areas by the interslice distance. Bars = 20 µm

The C-SNARF-4 images were then imported into the software daime (digital image analysis in microbial ecology, v. 2.2)^36^ and segmented using intensity thresholding to remove microbial cells. The resulting green and red channel images were exported to ImageJ^37^ and divided by each other to obtain the fluorescence intensity ratios (r) for all extracellular areas. Finally, the average extracellular biofilm pH (±SD) in each FOV was calculated using the calibration Eq. (1). The average pH in each biofilm (9 FOVs) was determined and pH differences (ΔpH) between paired ARG and NoARG biofilms were calculated for each participant. The digital image analysis procedure is illustrated in Fig. 7.

### Fluorescence lectin-binding analysis (FLBA)

Matrix carbohydrate components in NoARG and ARG biofilms were visualized by FLBA^17^ using the fucose-binding lectin *Aleuria arantia* (AAL; SMS Gruppen, Rungsted Kyst, Denmark) and the galactose-binding lectin *Morus nigra* agglutinin G (MNA-G; SMS Gruppen), both labeled with fluorescein isothiocyanate (FITC).

Briefly, in situ-grown biofilms collected from each patient (*N* = 1 per lectin and intervention) were washed in phosphate-buffered saline (PBS, pH 7.4) and fixed in 3% paraformaldehyde for 3 h at 4 °C. After fixation, the biofilms were washed in PBS (3×, 5 min) and stored in a 50:50 (v/v) mix of PBS:ethanol until analysis. FLBA was performed by incubating the biofilms with 15 µL of the respective fluorescein isothiocyanate-labeled lectin (100 µmol/L) for 30 min at room temperature (RT). Thereafter, the biofilms were washed with PBS (3×) to remove unbound lectins, and counterstained with SYTO 60 (10 μmol/L; Molecular Probes, Thermo Fisher, Carlsbad, CA, USA) for 10 min (RT) to visualize microbial cells. Confocal microscopy images were acquired in the laser-marked FOVs as 6-sliced z-stacks spanning the height of the biofilms using an inverted confocal microscope (Zeiss LSM 700). The FITC-labeled lectins were excited at 488 nm and detected from 300-628 nm. SYTO60 was excited at 639 nm and detected from 644-800 nm. All images were obtained with a pixel dwell time of 2.5 µs and the pinhole set to 1.6 AU (1.3 µm optical slice). Laser power (488 nm) and detector gain were kept constant for each fluorescently labeled lectin and across all biofilm samples.

The biovolumes of microbial cells and lectin-targeted carbohydrates in each 6-sliced z-stack image were estimated quantified using the software daime,^36^ as described by Del Rey et al.^18^. Briefly, the areas covered by microorganisms (red channel) and carbohydrates (green channel) were identified in all images using intensity threshold-based segmentation. The biovolume of microorganisms and carbohydrate matrix components in the bottom (slices 1-2), middle (slices 3-4) and top (slices 5–6) layers of the biofilms, as well as the total biovolumes (slices 1–6), were estimated by multiplying the interslice distance with the respective areas covered by cells or lectin-targeted matrix components.^38^ To quantify carbohydrate matrix components only in the intercellular spaces, all procedures were performed after excluding the lectin-stained areas associated with microbial cells from the images. Normalized total and intercellular lectin-stained biovolumes were obtained by dividing the lectin-stained biovolumes by the respective microbial biovolumes. The digital image analysis procedures are shown in Fig. 5.

### DNA extraction and bacterial 16S rRNA sequencing

The bacterial composition of ARG and NoARG biofilms was analyzed by next-generation 16S rRNA gene amplicon sequencing. Biofilms from all participants (*N* = 1 per intervention) were collected, washed in PBS (1x) and stored in a PowerBead tube (Qiagen, Hilden, Germany) containing PowerBead Solution (Qiagen) at −20 °C. The DNA extraction was subsequently performed using the DNeasy PowerLyzer® PowerSoil®200 DNA Isolation Kit (Qiagen), according to the manufacturer´s instructions. The primers Bac 314 F and Bac 805R^39^ were used to amplify the V3-V4 region of bacterial 16S ribosomal RNA gene, as described by Tawakoli et al.^21^. Paired-end sequencing (2 × 300 bp) was performed on an Illumina MiSeq sequencer using the V3 sequencing kit (Illumina, Inc., San Diego, CA, USA). DNA extraction blanks (i.e. no sample was added during DNA extraction) and PCR negatives were included for sequencing. All sequence analyses were performed on R v. 4.4.1.^40^ Sequences were trimmed to remove barcodes and primers using cutadapt v. 0.2.0.^41^ Error correction, amplicon sequence variant (ASV) calling, chimera removal, and taxonomic classification were performed with the R package ‘DADA2’ v. 1.28.0.^42^ Classification was performed according to the Silva SSU reference database nr. 138.^43^ Nucleic acid extraction blanks and PCR negatives were used for decontaminating the data using the R package Decontam v. 1.20.0.^44^ Contaminants were found using the prevalence method with a threshold of 0.1 and subsequently removed from the data. A principal component analysis was carried out on centered log-ratio (clr) transformed read counts.

Relevant ASVs were identified using the Basic Local Alignment Search Tool (BLAST) of the expanded Human Oral Microbiome Database (http://www.homd.org) (Supplementary Table 2). ASV tables with taxonomic identification, relative abundance, and metadata can be found in Supplementary Table 3; raw sequence data are available at NCBI (BioProject PRJNA1156999).

### Statistical analysis

Differences in biofilm pH between ARG and NoARG, intragroup pH differences, and differences in lectin-stained biovolumes between groups were analyzed using linear mixed-effects models, which accounted for the hierarchical structure of FOVs nested within biofilms and biofilms nested within patients. The relationship between average biofilm pH and lectin-stained biovolumes at the patient level was analyzed using simple linear regression models. Likewise, the relationship between differences in pH (ΔpH) and in lectin-stained biovolumes (Δbiovolume) between ARG and NoARG biofilms at the patient level was analyzed with simple linear regression models. Coefficients of variation were used to quantify the variability in pH and lectin-stained biovolume across different FOVs within the same biofilm. Differences in biofilm thickness between groups were compared using paired t-tests. Differentially abundant ASVs in paired biofilm samples were identified using Wilcoxon signed-rank tests on clr-transformed read counts. The *p*-values were adjusted for false discovery rate using the Benjamini & Hochberg method. Statistical analyses were performed on R v. 4.3.0^40^ and GraphPad Prism v. 10 (GraphPad Software Inc., San Diego, CA, USA) with a significance level of α = 0.05.

## Supplementary information

Supplementary Table 3

Supplementary Table 6

Supplementary material

## Data Availability

The data generated during this study are available within the article and its supplementary files. Supplementary information accompanies the manuscript on the International Journal of Oral Science website http://www.nature.com/ijos. Sequence files and metadata for all samples are also publicly available under the Bioproject ID PRJNA1156999.
