# Supplementary material for: Arginine modulates the pH, microbial composition, and matrix architecture of biofilms from caries-active patients
Source: Int J Oral Sci. 2025 Nov 20;17:70. doi: 10.1038/s41368-025-00404-5 (PMC12630650; doi:10.1038/s41368-025-00404-5)
Supplement: Supplementary file 3 — Supplementary material [file 41368_2025_404_MOESM3_ESM.pdf]

Supplementary Figures

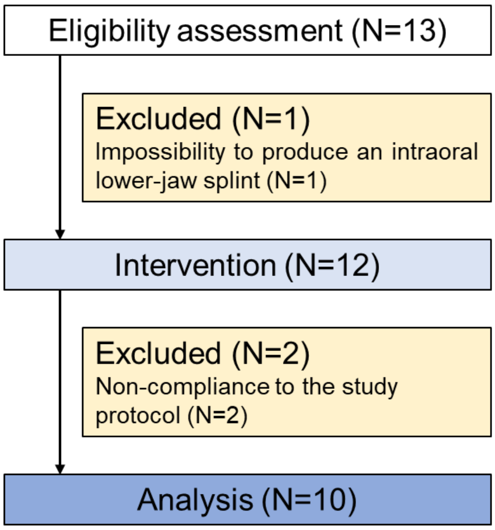

Supplementary Figure 1. CONSORT flow diagram.

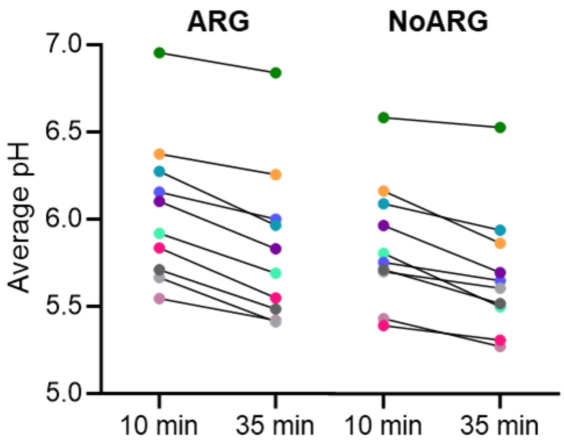

Supplementary Figure 2. Biofilm pH response after 10 and 35 min of sucrose challenge at the individual level. The colored dots represent the average pH of duplicate arginine- (ARG) or placebo-treated (NoARG) biofilms from each caries-active patient (N=10). Overall, arginine treatment increased the resilience of the biofilms to sucrose-induced pH drops. Only two patients, represented by the black and gray dots, exhibited a lower pH in ARG compared to NoARG biofilms.

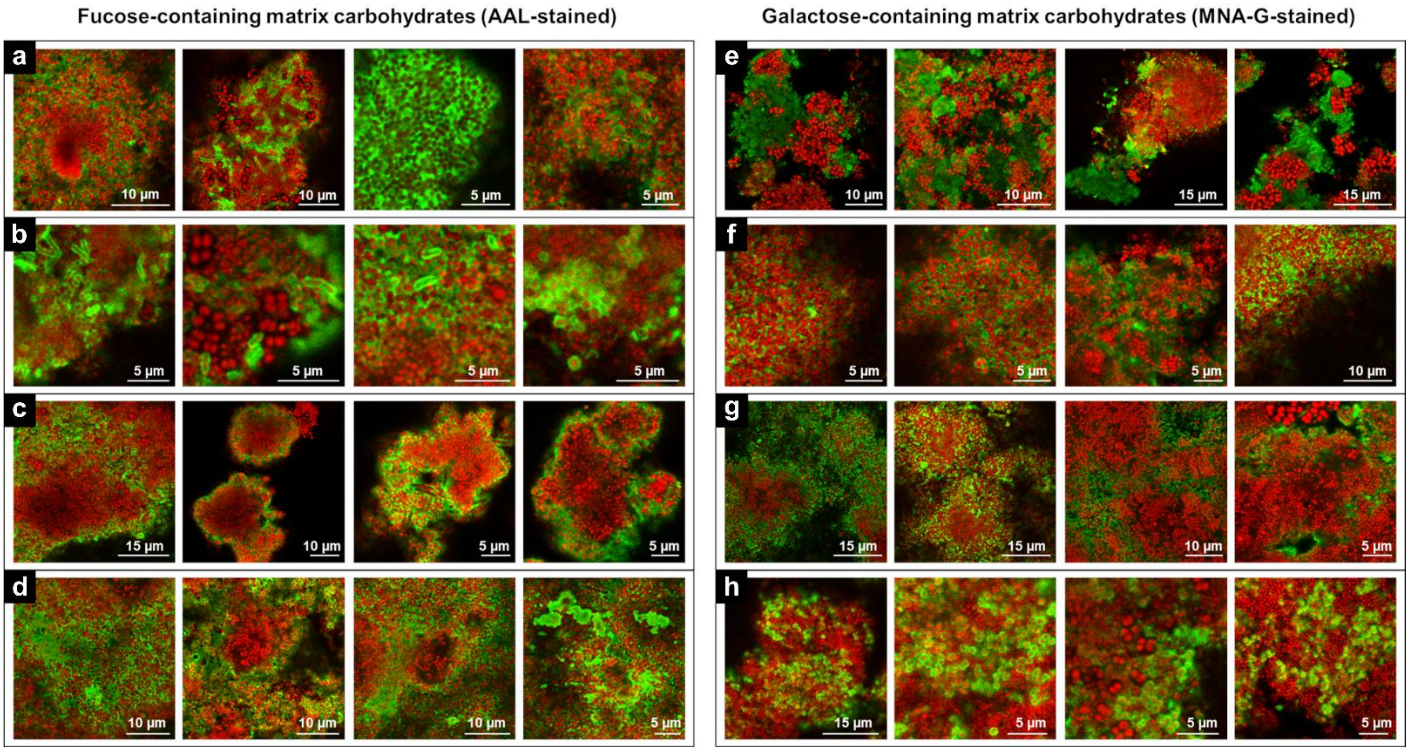

Supplementary Figure 3. Binding patterns of the lectins AAL and MNA-G in 4-day old *in situ*-grown biofilms from caries-active patients. Panels **a-d** show typical binding patterns of the fucose-specific lectin AAL. AAL mainly targeted glycoconjugates/polysaccharides located inside dense bacterial clusters, often binding to the surface of microbial cells (**a-b**). AAL also targeted carbohydrates in the surface layer of bacterial clusters, often extending to cell-free areas of the biofilm (**c-d**). All patterns were observed in both arginine- (ARG) and placebo-treated (NoARG) biofilms. Panels **e-h** show binding patterns of the galactose-specific lectin MNA-G. MNA-G frequently bound to cell-free areas in the biofilms (**e**), but it also visualized areas inside (**f**) or at the surface of bacterial clusters (**g**). In one patient, MNA-G targeted the surface of specific microbial cells (**h**). All patterns were observed in both ARG and NoARG biofilms.

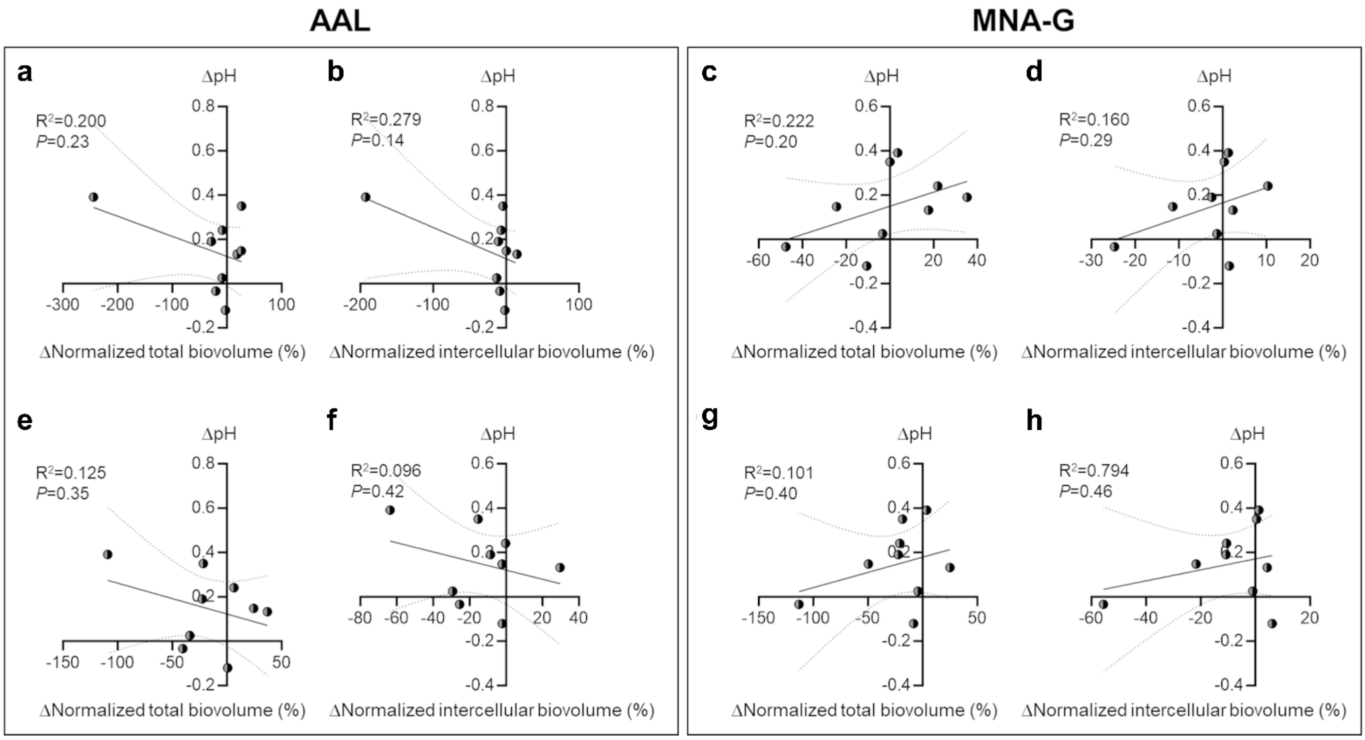

Supplementary Figure 4. Relationship between the abundance of carbohydrate matrix components and the effect of arginine on biofilm pH. a-d) Linear regression analyses showed no significant relationship between the paired differences in biofilm pH (ΔpH, ARG-NoARG) and paired biovolume differences (Δnormalized biovolumes, ARG-NoARG) in total or intercellular lectin-stained matrix components visualized in the entire biofilm biovolume. **e-h)** No significant relationship was found between ΔpH and Δtotal or Δintercellular lectin-stained biovolumes visualized at the biofilm bottom. Dots represent paired values for each caries-active patient (N=9 patients). Positive values indicate higher pH/biovolumes in ARG biofilms; negative values indicate higher pH/biovolumes in NoARG biofilms. AAL: left box, MNA-G: right box.

Supplementary Tables

Supplementary Table 1. Clinical status of the caries-active patients included in the analysis (N=10).

Number of active cavitated lesions	5.6 ± 3.5
Number of active non-cavitated lesions	3.4 ± 2.5
Total number of active lesions	9.9 ± 5.5

Supplementary Table 2. Reference 16S rRNA sequences of the extended Human Oral Microbiome Database (eHOMD, http://www.homd.org) with 100% alignment (427/427 nucleotide bases) with relevant amplicon sequence variants (ASVs).

	ASV sequence	Reference	Species
Streptococcus ASV1	TAGGGAATCTTCGGCAATGGACGGAAGTCTGACCGAGCAACGCCGCGTGAG<br>TGAAGAAGGTTTTCGGATCGTAAAGCTCTGTTGTAAGAGAAGAACGAGTGTGA<br>GAGTGGAAAGTTCACACTGTGACGGTATCTTACCAGAAAGGGACGGCTAACT<br>ACGTGCCAGCAGCCGCGGTAATACGTAGGTCCCGAGCGTTGTCCGGATTTAT<br>TGGGCGTAAAGCGAGCGCAGGCGGTTAGATAAGTCTGAAGTTAAAGGCTGTG<br>GCTTAACCATAGTACGCTTTGGAAACTGTTTAACTTGAGTGCAAGAGGGGAGA<br>GTGGAATTCCATGTGTAGCGGTGAAATGCGTAGATATATGGAGGAACACCGG<br>TGGCGAAAGCGGCTCTCTGGCTTGTAACTGACGCTGAGGCTCGAAAGCGTG<br>GGGAGCAAACA	058BW009	*Streptococcus oralis* subsp._dentisani_clade_058 (HMT-058), clone BW009
		058BM035	*Streptococcus oralis* subsp._dentisani_clade_058 (HMT-058), clone BM035
		058_8632	*Streptococcus oralis* subsp._dentisani_clade_058 (HMT-058), clone C3MLM097
		071_8234	*Streptococcus oralis* subsp._tigurinus_clade_071 (HMT-071), strain AZ_3a
		071_8631	*Streptococcus oralis* subsp._tigurinus_clade_071 (HMT-071),clone C3ALM006
		071_7062	*Streptococcus oralis* subsp._tigurinus_clade_071 (HMT-071), clone P4PA_13
		061DN025	*Streptococcus* sp._HMT_061 (HMT-061), clone: DN025
		707_3932	*Streptococcus* oralis subsp._oralis (HMT-707), strain ATCC 35037
		070-7A	*Streptococcus* oralis subsp._tigurinus_clade_070 (HMT-070), strain Hans 7A
		070-12F	*Streptococcus oralis* subsp._tigurinus_clade_070 (HMT-070), strain Hans 12F
		677_3929	*Streptococcus mitis* (HMT-677) , strain ATCC 49456
		423_8630	*Streptococcus* sp._HMT_423 (HMT-423), clone C2MKM128
		064_8609	*Streptococcus* sp._HMT_064 (HMT-064), clone C5MLM037
		431_8633	*Streptococcus infantis* clade_431 (HMT-431), clone C4AKM023
		638_8315	*Streptococcus infantis* clade_638 (HMT-638), strain ATCC 700779
		398_5045	*Streptococcus oralis* subsp._dentisani_clade_398 (HMT-398), strain SK34
		398_7051	*Streptococcus oralis* subsp._dentisani_clade_398 (HMT-398), clone P2PA_41
Streptococcus ASV3	TAGGGAATCTTCGGCAATGGGGGCAACCCTGACCGAGCAACGCCGCGTGAG<br>TGAAGAAGGTTTTCGGATCGTAAAGCTCTGTTGTAAGAGAAGAACGAGTGTGA<br>GAGTGGAAAGTTCACACTGTGACGGTAACTTACCAGAAAGGGACGGCTAACT<br>ACGTGCCAGCAGCCGCGGTAATACGTAGGTCCCGAGCGTTATCCGGATTTAT<br>TGGGCGTAAAGCGAGCGCAGGCGGTTAGATAAGTCTGAAGTTAAAGGCTGTG<br>GCTTAACCATAGTACGCTTTGGAAACTGTTTAACTTGAGTGCAGAAGGGGAGA<br>GTGGAATTCCATGTGTAGCGGTGAAATGCGTAGATATATGGAGGAACACCGG<br>TGGCGAAAGCGGCTCTCTGGTCTGTAACTGACGCTGAGGCTCGAAAGCGTG<br>GGGAGCAAACA	411_9017	*Streptococcus parasanguinis* clade_411 (HMT-411), clone SJTU_F_10_28
		411_8629	*Streptococcus parasanguinis* clade_411 (HMT-411), clone C2MKM006
		411_8634	*Streptococcus parasanguinis* clade_411 (HMT-411), clone C4MKM110
Neisseria ASV4	TGGGGAATTTTGGACAATGGGCGCAAGCCTGATCCAGCCATGCCGCGTGTCT<br>GAAGAAGGCCTTCGGGTTGTAAAGGACTTTTGTCAGGGAAGAAAAGGGCGG<br>GGTTAATACCCCTGTCTGATGACGGTACCTGAAGAATAAGCACCGGCTAACTA<br>CGTGCCAGCAGCCGCGGTAATACGTAGGGTGCGAGCGTTAATCGGAATTACT<br>GGGCGTAAAGCGGGCGCAGACGGTTACTTAAGCAGGATGTGAAATCCCCGG<br>GCTCAACCTGGGAACTGCGTTCTGAACTGGGTGACTAGAGTGTGTCAGAGGG<br>AGGTAGAATTCCACGTGTAGCAGTGAAATGCGTAGAGATGTGGAGGAATACC<br>GATGGCGAAGGCAGCCTCCTGGGATAACACTGACGTTCATGCCCGAAAGCGT<br>GGGTAGCAAACA	609_9301	*Neisseria flava* (HMT-609), strain U40
		764_9292	*Neisseria sicca* (HMT-764), strain Q13
		099N064A	*Neisseria macacae* (HMT-099), strain M-740
		682_9282	*Neisseria mucosa* (HMT-682), strain LNP405
Haemophilus ASV5	TGGGGAATATTGCGCAATGGGGGCAACCCTGACGCAGCCATGCCGCGTGAA<br>TGAAGAAGGCCTTCGGGTTGTAAAGTTCTTTCGGTAGCGAGGAAGGCATTTA<br>GTTTAATAGACTAGGTGATTGACGTTAACTACAGAAGAAGCACCGGCTAACTC<br>CGTGCCAGCAGCCGCGGTAATACGGAGGGTGCGAGCGTTAATCGGAATAAC<br>TGGGCGTAAAGGGCACGCAGGCGGTGACTTAAGTGAGGTGTGAAAGCCCCG	718N000A	*Haemophilus parainfluenzae* (HMT-718), clone ncd81b03c1
		718_3530	*Haemophilus parainfluenzae* (HMT-718), strain CIP 102513

	GGCTTAACCTGGGAATTGCATTTCATACTGGGTCGCTAGAGTACTTTAGGGAG<br>GGGTAGAATTCCACGTGTAGCGGTGAAATGCGTAGAGATGTGGAGGAATACC<br>GAAGGCGAAGGCAGCCCCTTGGGAATGTACTGACGCTCATGTGCGAAAGCG<br>TGGGGAGCAAACA		
Fusobacterium ASV7	TGGGGAATATTGGACAATGGACCAAAAGTCTGATCCAGCAATTCTGTGTGCAC<br>GATGACGTTTTTCGGAATGTAAAGTGCTTTCAGTTGGGAAGAAAAAAATGACG<br>GTACCAACAGAAGAAGTGACGGCTAAATACGTGCCAGCAGCCGCGGTAATAC<br>GTATGTCACAAGCGTTATCCGGATTTATTGGGCGTAAAGCGCGTCTAGGTGG<br>TTATGTAAGTCTGATGTGAAAATGCAGGGCTCAACTCTGTATTGCGTTGGAAA<br>CTGCATGACTAGAGTACTGGAGAGGTAAGCGGAACTACAAGTGTAGAGGTGA<br>AATTCGTAGATATTTGTAGGAATGCCGATGGGGAAGCCAGCTTACTGGACAG<br>ATACTGACGCTAAAGCGCGAAAGCGTGGGTAGCAAACA	201BS011	*Fusobacterium periodonticum* (HMT-201), clone BS011
Streptococcus ASV8	TAGGGAATCTTCGGCAATGGACGAAAGTCTGACCGAGCAACGCCGCGTGAGT<br>GAAGAAGGTTTTCGGATCGTAAAGCTCTGTTGTAAGAGAAGAACGGGTGTGA<br>GAGTGGAAAGTTCACACTGTGACGGTATCTTACCAGAAAGGGACGGCTAACT<br>ACGTGCCAGCAGCCGCGGTAATACGTAGGTCCCGAGCGTTGTCCGGATTTAT<br>TGGGCGTAAAGCGAGCGCAGGCGGTTAGATAAGTCTGAAGTTAAAGGCTGTG<br>GCTTAACCATAGTACGCTTTGGAAACTGTTTAACTTGAGTGCAGAAGGGGAGA<br>GTGGAATTCCATGTGTAGCGGTGAAATGCGTAGATATATGGAGGAACACCGG<br>TGGCGAAAGCGGCTCTCTGGTCTGTAACTGACGCTGAGGCTCGAAAGCGTG<br>GGGAGCGAACA	622_3931	*Streptococcus gordonii* (HMT-622), strain ATCC 10558
Streptococcus ASV9	TAGGGAATCTTCGGCAATGGGGGCAACCCTGACCGAGCAACGCCGCGTGAG<br>TGAAGAAGGTTTTCGGATCGTAAAGCTCTGTTGTAAGTCAAGAACGAGTGTGA<br>GAGTGGAAAGTTCACACTGTGACGGTAGCTTACCAGAAAGGGACGGCTAACT<br>ACGTGCCAGCAGCCGCGGTAATACGTAGGTCCCGAGCGTTGTCCGGATTTAT<br>TGGGCGTAAAGCGAGCGCAGGCGGTTTGATAAGTCTGAAGTTAAAGGCTGTG<br>GCTCAACCATAGTTCGCTTTGGAAACTGTCAAACTTGAGTGCAGAAGGGGAG<br>AGTGGAATTCCATGTGTAGCGGTGAAATGCGTAGATATATGGAGGAACACCG<br>GTGGCGAAAGCGGCTCTCTGGTCTGTAACTGACGCTGAGGCTCGAAAGCGT<br>GGGGAGCGAACA	755N000A<br>755_8839<br>021_8353	*Streptococcus salivarius* (HMT-755), strain ATCC 7073<br>*Streptococcus salivarius* (HMT-755), strain ATCC 13419<br>*Streptococcus vestibularis* (HMT-021), strain ATCC 49124
Streptococcus<br>ASV16	TAGGGAATCTTCGGCAATGGACGGAAGTCTGACCGAGCAACGCCGCGTGAG<br>TGAAGAAGGTTTTCGGATCGTAAAGCTCTGTTGTAAGAGAAGAACGAGTGTGA<br>GAGTGGAAAGTTCACACTGTGACGGTAACTTACCAGAAAGGGACGGCTAACT<br>ACGTGCCAGCAGCCGCGGTAATACGTAGGTCCCGAGCGTTATCCGGATTTAT<br>TGGGCGTAAAGCGAGCGCAGGCGGTTAGATAAGTCTGAAGTTAAAGGCTGTG<br>GCTTAACCATAGTACGCTTTGGAAACTGTTTAACTTGAGTGCAGAAGGGGAGA<br>GTGGAATTCCATGTGTAGCGGTGAAATGCGTAGATATATGGAGGAACACCGG<br>TGGCGAAAGCGGCTCTCTGGTCTGTAACTGACGCTGAGGCTCGAAAGCGTG<br>GGGAGCAAACA	721_3933<br>066FN051	*Streptococcus parasanguinis* clade_721 (HMT-721), strain ATCC 15912<br>*Streptococcus sp.*_HMT_066 (HMT-066), clone FN051

Supplementary Table 3 (available as .xlsx file). Read counts and relative abundances of the detected amplicon sequence variants (ASVs), results of the paired analysis of differentially abundant ASVs and genera (Wilcoxon signed-rank test, false discovery rate-adjusted P-values threshold of <0.05), and relevant associated sample metadata.

Supplementary Table 4. Average variation (±SD) in pH and normalized total lectin-stained biovolumes between fields of view of the same biofilm, for both arginine- and placebo-treated groups.

	**Arginine**	**Placebo**
pH (10 min)	1.5% ± 1.1%	1.7% ± 1.1%
pH (35 min)	1.6% ± 1.2%	1.9% ± 1.5%
AAL	46.2% ± 43.6%	54.5% ± 33.6%
MNA-G	70.0% ± 53.2%	49.1% ± 28.7%

Supplementary Table 5. Spearman rank correlations of the effect of arginine on biofilm pH and on the abundance of relevant genera and amplicon sequence variants (ASVs). Data used for the correlative analyses were the paired final pH differences between arginine- (ARG) and placebo-treated (NoARG) biofilms, and the paired differences (ARG-NoARG) in mean relative abundances of each genera/ASV.

**Genus**	**ASV**	**Rho**	**False discovery rate-adjusted *P*-value**
Streptococcus		-0.20	0.971
Veillonella		-0.16	0.971
Haemophilus		-0.09	0.971
Fusobacterium		0.08	0.971
Neisseria		-0.14	0.971
Rothia		0.39	0.971
Granulicatella		-0.55	0.971
Streptococcus	ASV1	0.07	0.892
Streptococcus	ASV9	0.08	0.892
Streptococcus	ASV3	-0.12	0.892
Streptococcus	ASV8	-0.09	0.892
Streptococcus	ASV16	0.18	0.892
Fusobacterium	ASV7	0.18	0.892
Haemophilus	ASV5	-0.58	0.892
Neisseria	ASV4	0.10	0.892

Supplementary Table 6 (available as .xlsx file). Fluorescence lectin-binding analysis of biofilms treated with arginine (ARG) or placebo (NoARG) using the lectins AAL (fucose-binding) or MNA-G (galactose-binding).
